# Long-Term Care Home Size Association with COVID-19 Infection and Mortality in Catalonia in March and April 2020

**DOI:** 10.3390/epidemiologia3030029

**Published:** 2022-09-05

**Authors:** Maria Victoria Zunzunegui, François Béland, Manuel Rico, Fernando J. García López

**Affiliations:** 1 École de Santé Publique, Université de Montréal, Montreal, QC H3N 1X9, Canada; 2 Institut Lady Davis, Montreal Jewish Hospital, McGill University, Montreal, QC H3C 3J7, Canada; 3 Infolibre, 28010 Madrid, Spain; 4 National Epidemiology Centre, Carlos III Health Institute, 28029 Madrid, Spain

**Keywords:** long-term care, COVID-19 mortality, COVID-19 incidence, risk factors

## Abstract

We aim to assess how COVID-19 infection and mortality varied according to facility size in 965 long-term care homes (LTCHs) in Catalonia during March and April 2020. We measured LTCH size by the number of authorised beds. Outcomes were COVID-19 infection (at least one COVID-19 case in an LTCH) and COVID-19 mortality. Risks of these were estimated with logistic regression and hurdle models. Models were adjusted for county COVID-19 incidence and population, and LTCH types. Sixty-five per cent of the LTCHs were infected by COVID-19. We found a strong association between COVID-19 infection and LTCH size in the adjusted analysis (from 45% in 10-bed homes to 97.5% in those with over 150 places). The average COVID-19 mortality in all LTCHs was 6.8% (3887 deaths) and 9.2% among the COVID-19-infected LTCHs. Very small and large homes had higher COVID-19 mortality, whereas LTCHs with 30 to 70 places had the lowest level. COVID-19 mortality sharply increased with LTCH size in counties with a cumulative incidence of COVID-19 which was higher than 250/100,000, except for very small homes, but slightly decreased with LTCH size when the cumulative incidence of COVID-19 was lower. To prevent infection and preserve life, the optimal size of an LTCH should be between 30 and 70 places.

## 1. Introduction

Since the beginning of the pandemic, almost 35,000 COVID-19 deaths in long-term care homes (LTCHs) in Spain have occurred and it has become clear that a major transformation of the long-term care model is urgently needed [1,2,3]. This is a study of the COVID-19 pandemic infection and mortality management in March and April 2020 by LTCHs licensed in Catalonia, the region of Spain with the largest number of LTCHs and the largest population of LTCH residents [4].

In Catalonia, as in other regions of Spain and in other European countries, multinational corporations have invested heavily in the long-term care sector over the last two decades, building large facilities of often over 100 places [5]. Few publicly owned and administered LTCHs are currently run by the Generalitat de Catalunya, the Government of Catalonia, henceforth referred to as the Generalitat [6]. At present, long-term care for older adults in Catalonia is characterised by institutions operating under public-private partnership agreements or by completely private LTCHs. In addition to the Generalitat, municipal governments play a key role in long-term care in Catalonia; they operate through foundations in partnership with the nonprofit sector, including religious orders and cooperatives, or, less frequently, with for-profit enterprises.

The international literature on the COVID-19 pandemic in long-term care institutions has identified LTCH size as one of the highest risk factors for the entry of SARS-CoV-2 into the home and COVID-19 mortality [7,8,9,10,11,12] and even the strongest risk factor for COVID-19 outbreaks [13]. In Spain, several research papers and three official reports have identified facility size as a major risk factor for the entry of SARS-CoV2 into the home [14,15,16,17,18,19]. Evidence is weaker regarding the association between COVID-19 mortality and LTCH size. Some studies reported no association [20,21,22,23], others reported a significant positive association [11,24] and one study found a protective association probably attributable to new design features of LCTHs, including self-contained units of less than 40 places [25]. To guide current policy discussions, evidence is needed on how COVID-19 infection and mortality varied with LTCH size, and more specifically how size impacted the ability of LTCHs to avoid the introduction of SARS-CoV-2 and to protect residents’ lives during the first two months of the COVID-19 pandemic [26,27,28].

This paper aims to examine the association between COVID-19 infection and mortality and LTCH size in Catalonia in March and April 2020. The results are intended to guide public policy in the transition toward a model of care which is centered on the safety and well-being of older adults.

## 2. Materials and Methods

*Source of data.* All data on LTCHs in Catalonia was obtained from the Portal de Transparencia (Transparency Portal: https://governobert.gencat.cat/ca/transparencia/acces-informacio-publica/ (accessed on 17 June 2020)) a government office that provides access to information of public interest upon request. In the mortality analyses we included 965 of the 975 licensed LTCHs; 10 private nursing homes were excluded because their COVID-19 mortality data were missing.

*Outcomes*. We examine two outcomes: (1) a binary variable defined as the presence of at least one confirmed case of COVID-19 infection, hereafter *COVID-19 infection in the LTCH*; and (2) *COVID-19 mortality* calculated as the number of COVID-19 deaths, certified as a death of a COVID-19-positive person or a death that occurred in a person showing COVID-19 symptoms, divided by the number of places authorised to the LTCH.

*Explanatory variable*. The size of the LTCH was the main explanatory variable. In the absence of more precise information, we use the number of authorised places as an approximation of facility size. For the descriptive analysis, this variable was categorised by the number of places as follows: 0–49, 50–99, 100–149, and 150 and over.

*Confounders.* Potential confounding factors for the association between LTCH size and the two study outcomes were: the population of the county where the home was located [8,20,22], the county COVID-19 cumulative incidence rate (CIR) [16,22,23,29] and the LTCH type [20,22,25,30,31]. The county where the LTCH was located was available on the database accessed on the Transparency Portal. Data on county populations in January 2020 were downloaded from the Statistical Institute of Catalonia and used as a categorical variable for descriptive purposes and as a continuous variable in the statistical modelling analyses [32]. Monthly data on the average 14-day COVID-19 CIR per 100,000 inhabitants (CIR/100,000 inhabitants) for each county were obtained from the Statistical Institute of Catalonia [32]. The CIR average value for March and April 2020 was used as a continuous variable in the analyses and categorised for descriptive purposes.

*LTCH type* was defined by the combinations observed of ownership type (public, either Generalitat or municipal, or private, either for-profit or nonprofit) and administration type (type of public-private partnership, if any). These data were obtained from the Transparency Portal.

### Statistical Analyses

*Bivariate analyses.* Bivariate associations between COVID-19 infection and LTCH size, county population, county COVID-19 CIR and LTCH type were examined. The three potentially confounding variables (county population, county COVID-19 CIR, and LTCH type) were significantly associated with LTCH size and COVID-19 infection, and COVID-19 mortality in LTCHs with COVID-19 infection.

*Multivariate analyses.* The two outcome variables, *COVID-19 infection in the LTCH* and *COVID-19 mortality*, are associated in a three-step process. The design of this process takes into consideration Soldevila et al.’s [16] suggestions that the association of facility size with COVID-19 mortality may only be shown in LTCHs with COVID-19 infection. First, only some LTCHs were infected with COVID-19. Second, COVID-19 can occur only in LTCHs with COVID-19 infection. Third, some LTCHs infected with COVID-19 experienced no COVID-19 deaths but COVID-19 deaths can occur only in LTCHs with COVID-19 infection. COVID-19 mortality at each LTCH is a function of these three events.

Estimates of the associations between the two outcomes and LTCH size were obtained through regression procedures using appropriate linked functions which were adjusted by the confounders.

The distribution of the probability of COVID-19 infection at an LTCH was obtained with a regression equation using the logistic link function:

(1) $$P\left[I_{h}\right]=1/\left(1+e^{-\left(\propto_{I}+b_{1}S_{h}+\sum_{n}^{}c_{nI}F_{nh}\right)}\right)$$

with: $I_{h}=0\;:LTCHs\;without\;COVID19\;cases;$

and: $I_{h}=1\;:LTCHs\;with\;at\;least\;one\;COVID19\;case$;

where $P\left[I_{h}\right]$ is the probability of COVID-19 infection at an LTCH (hereafter, ‘*h*’); $\propto_{I}$ is the intercept, $b_{I1}$ is the regression coefficient for the size $S_{h}$ of the ‘*h*’. $F_{nh}$ stands for the n confounder for each of the ‘*h*’s, and $c_{nI}$ are the associated regression coefficients.

The distribution of mortality among LTCHs was asymmetrical. Almost half of the LTCHs (460/965) did not have any COVID-19 deaths. Also, no COVID-19 deaths were identified in 122 LTCHs out of the 627 with COVID-19 infection. These are excess zero cases in the distribution of COVID-19 deaths as in the Suñer et al. study [23]. These authors used zero-inflated Poisson models in their study of COVID-19 mortality in LTCHs. However, in Catalonia, some very small and some very large LTCHs had very few deceased residents while some had a large number. Thus, sub-groups of LTCHs may be characterized by a zero-deflation process. Where both zero-inflation and zero-deflation processes are observed, hurdle models performed better than zero-inflation models [33,34,35,36].

The hurdle and the zero-inflation models were tested. The parameter for the excess zero in the zero-inflation model was large and introduced problems in the estimation procedure, namely the singularity of the information matrix. This result suggests that the data generating process for the absence of COVID-19 deaths in LTCHs with COVID-19 infection were not the same as for the number of COVID-19 deaths. In this study, the association of LTCH size with the number of COVID-19 deaths in a COVID-19 infected LTCH was estimated by a hurdle model.

Hurdle models split the distribution of the number of COVID-19 deaths into two: the first part is concerned with LTCHs without COVID-19 deaths, and the second part examines LTCHs with at least one death [37,38].

For the first part of the hurdle model, the probability of an ‘*h*’ to have zero COVID-19 deaths in an ‘*h*’ with COVID-19 infection was obtained from a regression equation using the logistic link function:

(2) $$P\left[V_{h}\right]=1/(1+e^{-\left(\propto_{V}+b_{V}S_{h}+\sum_{n}c_{nV}F_{nh}\right)})$$

with $V_{h}=1:\;LTCHs\;without\;COVID19\;deaths$;

and $V_{h}=0\;:LTCHs\;with\;at\;least\;one\;COVID19\;death;$

(3) $$P\left[M_{h}\right]=1-P\left[V_{h}\right]$$

with $M_{h}=1:\;LTCHs\;with\;at\;least\;one\;COVID19\;death$;

and $M_{h}=0\;:LTCHs\;without\;COVID19\;death$;

where $P\left[V_{h}\right]$ is the probability of having zero COVID-19 deaths, while $P\left[M_{h}\right]$ is the probability of having at least one COVID-19 death. The remaining notation is as explained for Equation (1).

The second part of the hurdle model estimates the association of the size of an LTCH with the number of COVID-19 deaths in an LTCH with COVID-19 infection using the truncated negative binomial as the link function. The truncated Poisson link function within the hurdle model was also tested. However, using the likelihood ratio test, the AIC, and the BIC goodness of fit indexes, the null hypothesis of no differences in the estimated number of deaths was rejected (statistics not shown). Thus, the truncated negative binomial link function was retained in the hurdle model.

The number of COVID-19 deaths was estimated with the following equations obtained from a regression equation using the truncated negative binomial link function:

(4) $$Q\left[D_{h}\right]=e^{\left(\propto_{D}+b_{D}S_{hD}+\sum_{n}^{}c_{nD}F_{nhD}\right)}$$

with $D_{h}\geq 1\;:\;Number\;of\;deaths\;in\;the\;LTCH\;h$

$Q\left[D_{h}\right]$ is the number of COVID-19 deaths in an ‘*h*’. The remaining notation is as explained for Equation (2).

The second outcome in this research is obtained with the combination of Equations (1), (3) and (4). The proportion of residents dying from COVID-19 in an LTCH is the result of the combination of the probability of having at least one COVID-19 infection in that LTCH ($P\left[I_{h}\right])$, the probability of having at least one COVID-19 death in the LTCH ($P\left[M_{h}\right]),\;$ the number of COVID-19 deaths ($Q\left[D_{h}\right])$ and the quotient of the number of residents divided by LTCH size ($CD_{h}/R_{h}$) and *R_h_* is the number of residents in the LTCH.

(5) $$P\left[CD_{h}\right]=P\left[I_{h}\right]\times P\left[M_{h}\right]\times Q\left[D_{h}\right]\times CD_{h}/R_{h}$$

where $P\left[CD_{h}\right]$ is the proportion of COVID-19 deaths in the LTCH h.

The proportion of COVID-19 deaths in LTCHs with at least one COVID-19 infection ($P\left[CD_{h}\;|\;I_{h}=1\right]$) according to LTCH size can be obtained from:

(6) $$P\left[CD_{h}\;|\;I_{h}=1\right]=P\left[M_{h}\right]\times Q\left[D_{h}\right]\times CD_{h}/R_{h}$$

These equations were extended to include multiplicative interaction terms in the analyses based on the findings of the main effects models described above. All tables describing the statistical modelling results are shown in Appendix A, while the body of the paper contains the figures based on those models.

Analyses were carried out using IBM SPSS Statistics for Windows, version 23 (IBM Corp., Armonk, NY, USA) and MPlus version 8.5 (Muthèn & Muthèn, Los Angeles, CA, USA).

## 3. Results

LTCH occupancy in Catalonia was estimated to be close to 90% [39]. Fifty-two percent of LTCHs had less than 50 residents and 53% of all residents lived in those homes (Table 1). Half of the homes were located in the three counties in Catalonia with more than 700,000 inhabitants, which include the three major cities, and 47% of the residents lived in them. Twelve percent of the homes and 12% of LTCH residents in Catalonia were in counties with less than 100,000 inhabitants. More than one-third of the homes were located in counties with a very high COVID-19 CIR (Table 1).

**Figure 1:**
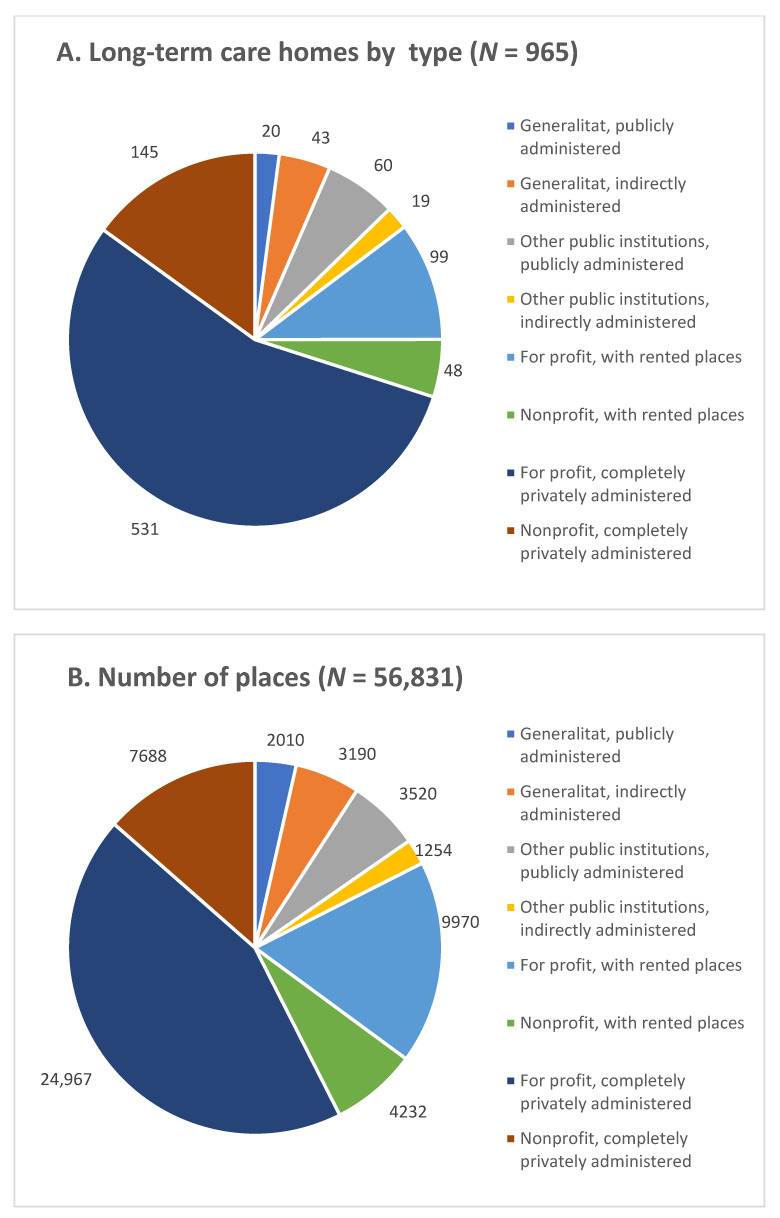
Distribution of LTCHs and number of places by LTCH type. (**A**) Number of LTCHs by type (**B**) Number of places by LTCH type.

The distributions of LCTHs by administrative type and number of places are shown in Figure 1. The most frequent type of public-private partnership (PPP) agreement between the public and private sectors involves the public sector renting a fixed number of places in private institutions and paying an amount to the private sector, regardless of their use or occupation (in the study period 147 LTCHs, 99 in the for-profit sector and 48 in the nonprofit sector had this type of agreement). A second form of PPP agreement is characterised by the public sector engaging a private enterprise to completely administer a publicly-owned home (in the study period, 43 LTCHs owned by the Generalitat and 19 owned by other public institutions fell into this category). There are other possible collaborations between the public and the private sector but they do not imply a partnership since the ownership and management are completely private. For example, the public sector finances a variable percentage of the cost of a place in a private LTCH while the elderly person waits for a vacancy in the public system. The proportion paid for by the state is called PEV, prestación económica vinculada in Spanish, or contribution-linked benefit.

For-profit privately administered homes were the most frequent, accounting for 55% of the homes and 44% of the residents. Publicly-owned and administered homes were the least frequent, with just 2% of the homes and 3% of the residents.

### 3.1. Bivariate Analyses: COVID-19 Infection as an Outcome

Overall, 627 (65%) of the 965 LTCHs in Catalonia were infected by COVID-19 in the two given months. The percentage of LTCHs with COVID-19 infection by LTCH size are shown in column 2 of Table 2. The percentage of COVID-19 infected LTCHs increased from 52% in homes with less than 30 places to 95% in those with 150–199 places.

The percentage of LTCHs with COVID-19 infection was largest in Barcelona (80%) and lower in those homes located in counties with less than 100,000 inhabitants (41%).

There was a linear relationship between the percentage of COVID-19 infected LTCHs and the COVID-19 CIR in the county where the home was located. In counties where the COVID-19 CIR was less than 500/100,000, 44% of LTCHs were infected by COVID-19; this figure was 65% in areas where the COVID-19 CIR was higher than 900/100,000.

The percentage of COVID-19 infected homes was higher in nonprofit LTCHs that rented places to the Generalitat (81%) or those owned by the Generalitat (75% and 77%) than in for-profit or nonprofit completely private LTCHs (61% and 64%, respectively) or in those administered by public institutions other than those directly administered by the Generalitat (58%).

### 3.2. Bivariate Analyses: COVID-19 Mortality as an Outcome

There were 3887 confirmed or suspected COVID-19 deaths in all LTCHs in March and April 2020. Dividing this figure by the maximum capacity of 56,831 places in the 965 LTCHs yields an observed COVID-19 mortality of 6.8%. Observed mortality in the 627 LTCHs with COVID-19 infection (with a total of 42,175 places) was 9.2%.

The percentage of LTCHs with at least one COVID-19 death categorised by size, county population, and county COVID-19 CIR and LTCH type is shown in column 3 of Table 2. Overall, the percentage of homes with at least one death was 52%.The percentage of LTCHs with at least one COVID-19 death increased from 38% in homes with less than 30 places to 90% for those with 150–199 places. The figure was larger in Barcelona’s LTCHs (69%) than in LTCHs in counties with less than 100,000 inhabitants (26%) and was largest in LTCHs located in counties where the CIR was higher than 900/100,000. Lastly, this figure was largest in the homes owned and administered by the Generalitat (70%) and in the nonprofit LTCHs renting places to the public sector.

The average number of COVID-19 deaths per LTCH was 7.7. This number increased with LTCH size from 3.2 in the LTCHs with less than 30 places to 30.3 in the LTCHs with more than 200 places. Out of the total of 3887 COVID-19 deaths occurring in the LTCHs in Catalonia during March and April 2020, 9% occurred in the 298 homes with less than 30 places and 24% occurred in the 42 homes with 150 or more places.

In addition, the number of deaths increased with the population of the county where the home was located and with the county COVID-19 CIR. It was largest in LTCHs with rented places and in LTCHs publicly owned and administered by the Generalitat, and lowest in completely private homes (Table 2).

### 3.3. Multivariate Analyses: COVID-19 Infection as an Outcome

The results of the logistic model for the association between COVID-19 infection and LTCH size, modelled as in equation 1, are shown in Table A1 (Appendix A). COVID-19 infection in LTCHs was a function of LTCH size in the unadjusted logistic regression and in the adjusted models, where only population size and county COVID-19 CIR were retained as confounders.

**Figure 2:**
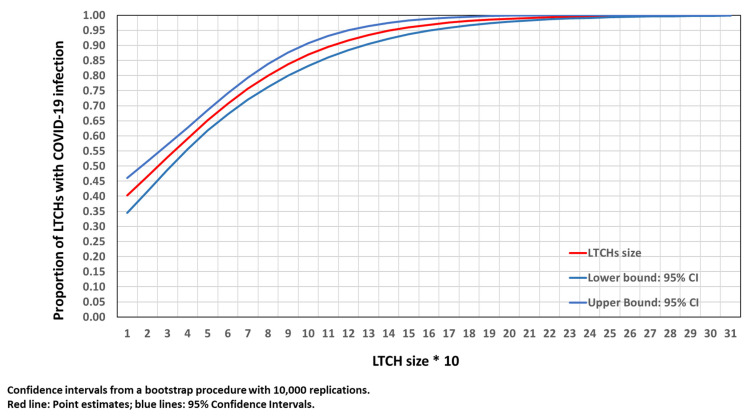
Proportion of LTCHs with COVID-19 infection by LTCH size.

In Figure 2, it can be seen that the likelihood of an LTCH to become infected by COVID-19 increased with its size. It doubled (from 0.38 to 0.75) when comparing LTCHs with 7 and 60 places. In LTCHs with 140 places, 95% of them were infected with COVID-19. The confidence intervals were small over the whole distribution of LTCH size. All LTCHs with 230 places were infected with COVID-19.

### 3.4. Multivariate Analyses: Proportion of LTCHs with COVID-19 Mortality

The first part of the hurdle model predicts the probability for a LTCH to pertain to the ‘no event’ category, i.e., the absence of death in the LTCH. Results are shown in part 1 of Table A2 in Appendix A.

Of the three potential confounders, only the county COVID-19 CIR remained significant and contributed to the equation.

**Figure 3:**
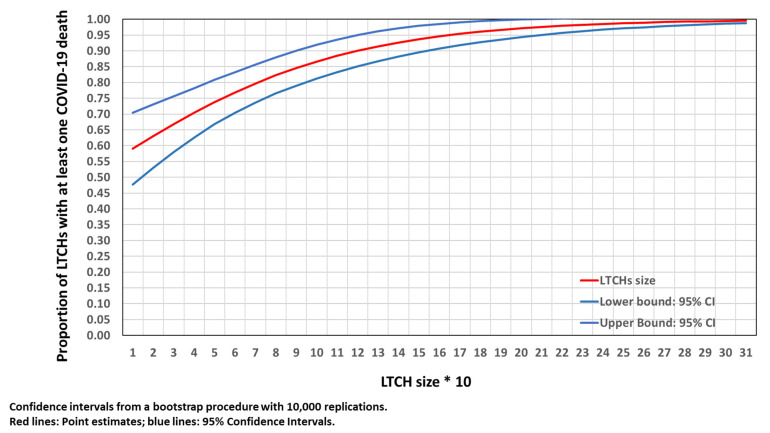
Proportion of LTCHs with at least one COVID-19 death by LTCH size.

Figure 3 shows the probability for a LTCH to have at least one COVID-19 death (Equation (3)). Smaller LTCHs with COVID-19 infection were less likely to have COVID-19 deaths. In homes with 70 places, the probability of having at least one COVID-19 death was 80%. This figure increased to 95% in homes with 160 places. The confidence intervals were small over the whole distribution of LTCH size. Almost all LTCHs with over 160 places experienced COVID-19 deaths.

### 3.5. Multivariate Analyses: Number of COVID-19 Deaths in LTCHs

The estimated number of COVID-19 deaths by LTCHs size was obtained from the second part of the hurdle model (Equation (4)). Discrepancies occurred between the estimated and the observed number of deaths. We examined the strength of the association between COVID-19 mortality with LTCH size according to several values of county COVID-19 CIR. Among counties with COVID-19 CIR with less than 250/100,000, observed COVID-19 mortality did not change with LTCH size, whereas among counties with COVID-19 CIR between 250 and 500/100,000, COVID-19 mortality increased slightly with LTCH size. Furthermore, among counties with COVID-19 CIR greater than 500/100,000 CIR, COVID-19 trends by LTCH size increased steeply. The results of these exploratory stratified analyses suggested that county COVID-19 CIR may shape the association between COVID-19 mortality and LTCH size, a modification that could be modelled by a multiplicative interaction term between LTCH size and county CIR for one of the main two outcomes of this paper, COVID-19 mortality. As county CIR was positively associated with county population, the interactions of county CIR and population size with LTCH size were both tested. Exploratory stratified analyses by county CIR were run on the distributions of observed proportions of LTCHs with COVID-19 infection and of LTCHs with at least one COVID-19 death by LTCH size. No evidence for interactions were found.

Results on the association of the number of COVID-19 deaths in LTCHs with LTCH size are reported first at the average county COVID-19 CIR in counties with LTCHs with COVID-19 infection (823/100,000) and second, variations of this association are examined at different levels of county COVID-19 CIR.

### 3.6. Number of COVID-19 Deaths in an LTCH by LTCH Size at the Average County COVID-19 CIR

**Figure 4:**
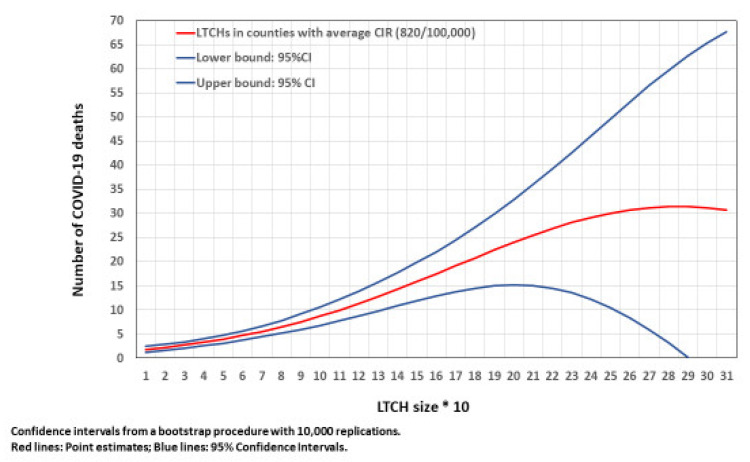
Number of COVID-19 deaths in an LTCH by LTCH size, for counties with CIR of 820/100,000.

Figure 4 was generated by Equation (4) (estimated number of COVID-19 deaths in an LTCH by LTCH size at 823/100,000). The number of COVID-19 deaths increased with LTCH size, reaching a maximum value of 270 places. Confidence intervals increased with LTCH size. Part of this increase may be attributed to the small number of LTCHs at the higher end of the distribution (only 9 LTCHs had more than 200 places). However, the continuously increasing confidence intervals with LTCH size reflected the observed distribution of COVID-19 deaths by LTCH size.

**Figure 5:**
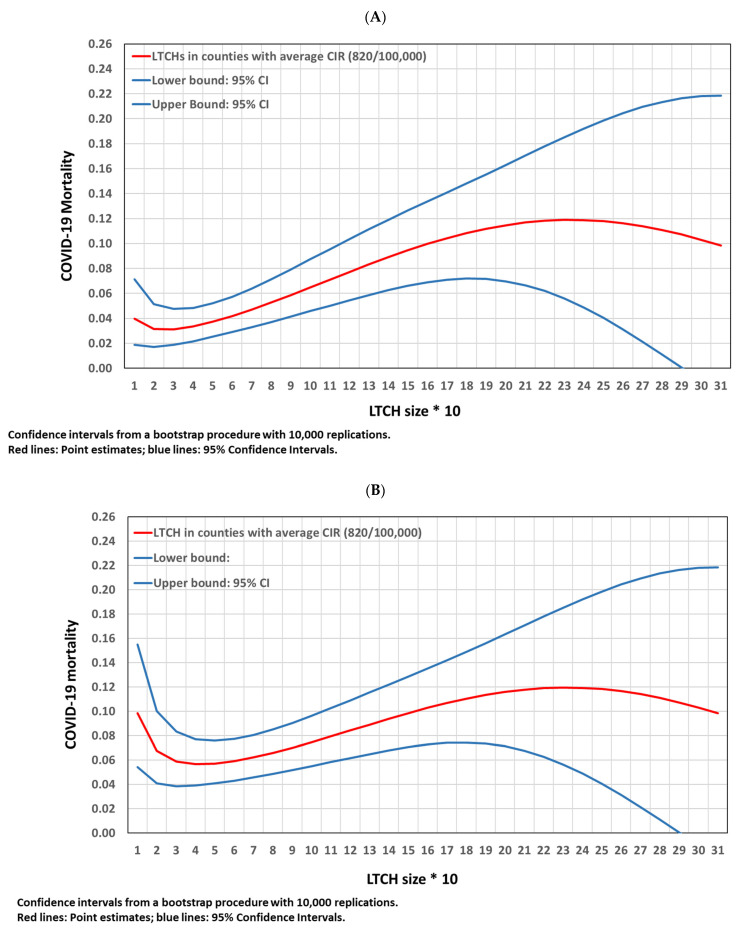
COVID-19 mortality proportion by LTCH size. (**A**) COVID-19 mortality proportion by LTCH size in all 965 LTCHs. (**B**) COVID-19 mortality by LTCH size in the 627 LTCHs with COVID-19 infection.

COVID-19 mortality proportion by LTCH size was obtained using Equation (5). Figure 5A shows COVID-19 mortality proportion by LTCH size in all 965 LTCHs. COVID-19 mortality in very small LTCHs (from 11 to 20 places) was higher than in LTCHs with 21 to 40 places. It reached 12% in LTCHs with 250 places and decreased to 10% for larger LTCHs. Confidence intervals (blue lines) were large for small and large LTCHs. Figure 5A shows COVID-19 mortality proportion by LTCH size.

Similar results were obtained, using Equation (6), for COVID-19 deaths in LTCHs with COVID-19 infection but with more dramatic figures for very small LTCHs (Figure 5B). Homes with less than 20 places had higher mortality than LTCHs with 50 places. However, confidence intervals were large in very small and very large LTCHs due to the small number of deaths in small LTCHs and the small number of very large LTCHs. LTCHs with 30 to 70 places had the lowest COVID-19 mortality (6%) in LTCHs with COVID-19 infection in Catalonia.

### 3.7. Number of COVID-19 Deaths in an LTCHs by LTCH Size Differed with County COVID-19 CIR Levels

**Figure 6:**
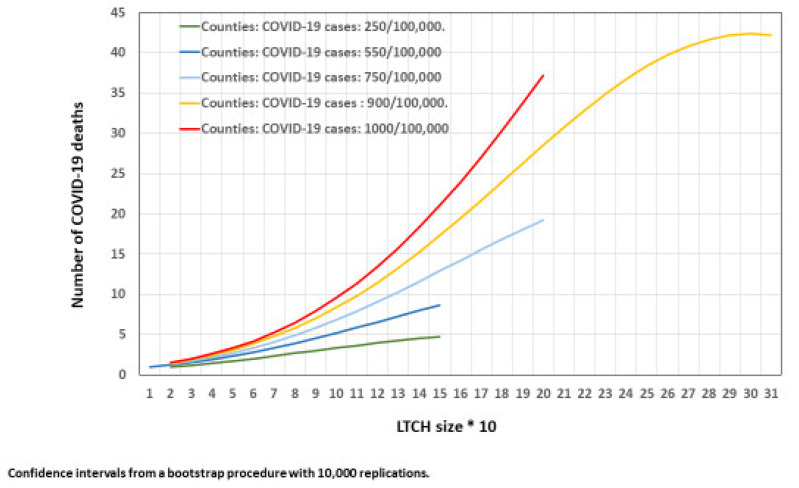
Number of COVID-19 deaths in LTCHs with at least one death by LTCH size at five county COVID-19 CIR levels.

Interactions of county CIR and population size with LTCH size were tested (see Appendix A) for the number of COVID-19 deaths (Part 2 of the hurdle model). The interaction term between county COVID-19 CIR and LTCH size was retained (Table A2, part 2 in Appendix A). In Figure 6, the curves represent the estimated number of COVID-19 deaths by LTCH size according to five values of county COVID-19 CIR. In a county with a COVID-19 CIR of 250/100,000, the number of COVID-19 deaths went from approximately one death in homes with 20 places to five deaths in homes with 150 places. For homes with 20 places, the number of estimated COVID-19 deaths in a county with a COVID-19 CIR of 1000/100,000 is similar to the estimated deaths for the smallest CIR of 250/100,000. However, in counties with COVID-19 CIR of 1000/100,000, there were 22 deaths in homes with 150 places, increasing to 37 for the homes with 200 places.

None of the nine LTCHs with more than 200 places were located in counties with a CIR higher than 900/100,000 and four LTCHs out of those nine were located in counties with a CIR between 750 and 900,000/100,000. Forty-three deaths per COVID-19 were estimated in LTCHs with 300 places. COVID-19 deaths plateaued after this number of places. Whether this pattern is reproducible in areas with a larger number of LTCHs in the four other CIR levels is an open question.

### 3.8. COVID-19 Mortality at an LTCH by LTCH Size According to Five Levels of County COVID-19 CIR

**Figure 7:**
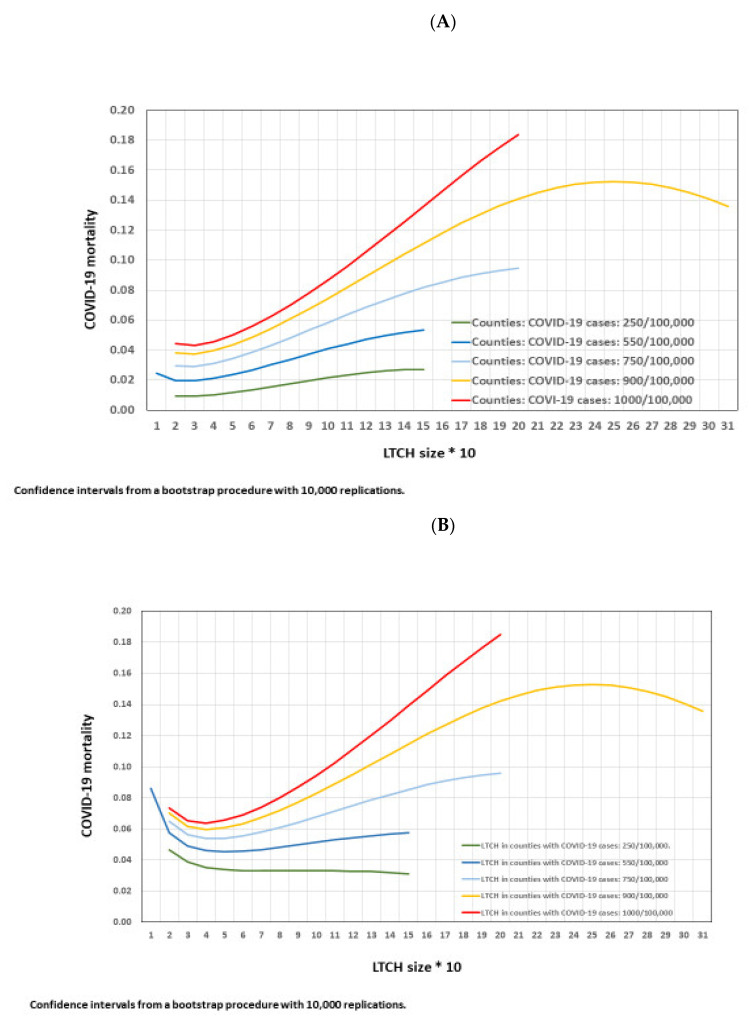
COVID-19 mortality by LTCH size at five county COVID-19 CIR levels. (**A**) In the 965 LTCHs (**B**) In the 625 LTCHs with COVID-19 infection.

The estimated number of deaths were used to compute COVID-19 mortality (Figure 7). In Figure 7A, the curves represent COVID-19 mortality at an LTCH (*n* = 965) by LTCH size according to five levels of county COVID-19 CIR. COVID-19 mortality increases with LTCH size at each increasing county COVID-19 CIR level and the curves are steeper as county COVID-19 CIR increases.

In Figure 7B, the curves represent COVID-19 mortality at LTCHs with COVID-19 infection (*n* = 625) by LTCH size at five county COVID-19 CIR levels. These curves follow similar patterns to the whole sample of 965 LTCHs but the curves are more pronounced at the lowest and highest ends of LTCH size.

Two notes of caution are needed in the interpretation of these results. First, COVID-19 mortality was larger in LTCHs with less than 30 places than in those with 40 places. The difference of one or two deaths in a home with 20 places adds a mortality risk of 10% or 20%. Second, estimations for homes larger than 200 places were available only for county COVID-19 CIR = 900/100,000 due to the small number of LTCHs with more than 200 places in Catalonia (*n* = 9).

## 4. Discussion

### 4.1. Summary of Results

Our results confirm that LTCH size was a strong risk factor for COVID-19 infection and mortality in Catalonia’s LTCHs. The percentage of homes infected by COVID-19 in March and April 2020 increased from 45% in LTCHs with 10 places to 85% in LTCHs with up to 90 places. The figures continue to increase, reaching 99% in LTCHs with 200 places, and peaking at 100% in the largest LTCHs. Adjusting by the confounders increased the strength of the association between COVID-19 infection and LTCH size.

In LTCHs with COVID-19 infection, size had a significant effect on mortality risk and on the probability of having at least one death. The percentage of LTCHs with at least one COVID-19 death increased linearly from 70% in an LTCH with 50 places to 90% in one with 100 places. Ninety-eight percent of LTCHs with 200 places had at least one COVID-19 death.

COVID-19 mortality figures were obtained at the average county COVID-19 CIR of 820/100,000 and, given the interaction of LTCH size with county COVID-19 CIR, for five levels of CIR. At the average county COVID-19 CIR, out of all 965 LTCHs, mortality was lowest in those with 11 to 50 places (from 3.5% to 4.3%). COVID-19 mortality in homes with 10 places was at 4%. Mortality increased from 5% to 12% in LTCH with 70 to 220 places. These homes seemed less able to protect their residents.

For LTCHs with COVID-19 infection, COVID-19 mortality was lower in LTCHs with 30 to 70 places (6% to 6.4%). In large LTCHs, COVID-19 mortality reached a maximum of 12%. Mortality at the level of 10% and over was observed in very small homes and in very large ones, though these results need to be interpreted with caution. As explained above, the difference of one or two deaths in a home with 20 places adds a mortality risk of 10% or 20%. It is also possible that these very small homes experienced more difficulties in preventing COVID-19 deaths at a time of general scarcity of diagnostic tests, and possible lack of staff, since the absence of one or two workers could be a substantial reduction of the staff during a critical period and a probable lack of the clinical abilities needed to confront catastrophic events.

Analyses of the interactions between LTCH size and county COVID-19 CIR on the number of COVID-19 deaths provided further insights into the impact of the general public health context during the pandemic. In counties with a COVID-19 CIR of 250/100,000, mortality showed a slight decrease with LTCH size, hovering at around 3%. In counties with a COVID-19 CIR of 550/100,000, COVID-19 mortality was high in the homes with less than 30 places, then decreased to 4.5% at 40 places and increased up to 5.7% at 140 places, which is the last data point available for estimation at this CIR value. In counties with a COVID-19 CIR of 1000/100,000, COVID-19 mortality increased from 6.2% at 40 places to 18% in homes with 200 places. The lowest mortality estimates were obtained in homes with a range of 30 to about 70 places at all levels of COVID-19 CIR.

### 4.2. Interpretation of Results Based on Available Literature

Soldevila et al. reported high COVID-19 infection risk in residents of large LTCHs in Barcelona and stated that large homes were more likely to have a carrier entering the LTCH given the large number of workers that entered the home every day [16]. Visitors could have transmitted the infection during visits in the first weeks of the pandemic, but were unlikely vectors during the strict lockdown ordered by the Government of Spain from 14 March to 11 May 2020. During most of the study period (March to April 2020), visits were cancelled and LTCH residents’ movement was restricted to their rooms. In a study of 9395 US-based nursing homes, Abrams et al., (2020) conclude that COVID-19 outbreaks were less frequent in LTCHs with 50 places or less, but a higher proportion of patients in small LTCHs were affected than in large LTCHs [40]. Our study suggests that in communities with low CIR, LTCH size is not related to COVID-19 mortality. Also, in communities with high CIR, mortality accelerates with LTCH size. Soldevilla et al. suggest that a higher number of workers and visitors in LTCHs may be related to increased COVID-19 infection [16]. The risk of transmission is lower in LTCHs located in counties with low CIR and higher in counties with high CIR, while LTCH size plays an accelerating role in COVID-19 transmission with the increase in the number of potential vectors of SARS-CoV-2.

The strong and significant association between LTCH size and COVID-19 mortality we report here is found in counties with a CIR greater than 250/100,000 and was not observed in a previous study conducted in Catalonia [23]. That study reported an association between LTCH size and all-cause mortality (but not with COVID-19 mortality) which could be related to policies enacted during the COVID-19 pandemic or to underdiagnoses of COVID-19 as a cause of death and it also reported that an increase of 100 in community COVID-19 CIR was associated with a 140% increase in LTCH mortality. In the same line of evidence on excess deaths in LTCHs linked to COVID-19, a study using administrative data on excess deaths in LTCHs in England showed that almost all excess deaths were registered in LTCHs that had COVID-19 deaths. In this study, (1) non-COVID deaths could have been misdiagnosed as COVID-19 deaths; and (2) indirect negative effects of COVID-19 were restricted to LTCHs with COVID-19 outbreaks [9]. The strong association between LTCH size and COVID-19 mortality in counties of high COVID-19 CIR could be explained by high infection rates in staff residing in close neighborhoods [41], potentially leading to staff reductions at the LTCHs due to sickness or absence or to an overload at local referral hospitals. However, we do not have the data necessary to examine these potential explanations.

As suggested by Soldevila et al. [16], features of the distribution of COVID-19 mortality appear more dramatically when only LTCHs with COVID-19 infection are considered. Also, mortality was higher in very small LTCHs than in medium-sized LTCHs. In effect, one death from COVID-19 in a 10-place facility gives a proportion of 10% deceased COVID-19 residents. However, this arithmetic operation alone cannot explain the variation in the proportions of COVID-19 deaths in LTCHs with less than 30 places.

This study adds to the literature on the association of COVID-19 mortality and LTCH size. As stated in the introduction, previous results have reported contradictory associations. Here, we have provided evidence that COVID-19 CIR in the counties where an LTCH is located shapes the association between COVID-19 mortality and LTCH size. These different findings may be explained by the characteristics of the communities where the LTCHs were located.

### 4.3. Study Limitations

First, this study did not include information on individual sociodemographic or health characteristics of LTCH residents. Frail, elderly residents may have been more exposed to multiple sources of infection because they require more intimate care and are more likely to be re-infected than similarly frail, older adults in smaller LTCHs, where staff and quality of care may have been more stable. Frail residents in large LTCHs may have been at a higher risk of mortality than similarly frail residents living in the smallest homes. The unavailability of individual characteristics did not allow us to test these hypotheses. Second, no information is available on LTCH characteristics known to be related to COVID-19 outcomes and that may be associated with the size of the facility, for instance, construction year, bathroom/resident ratio, crowding index, and staffing ratios. Third, the number of COVID-19-positive residents at each LTCH was available but not included in these analyses because it constitutes an underestimate of the spread of infection. During the first weeks of the epidemic, tests were generally not available and when available were only used to confirm the diagnosis in those with symptoms and to trace contacts. Fourth, there was an insufficient number of observations at the upper end of the LTCH size distribution to provide precise estimates for LTCHs with more than 200 places in the interaction analyses. Finally, discrepancies between observed COVID-19 mortality in LTCHs and mortality estimated with equation 4 led to the introduction of an interaction term between LTCH size and county COVID-19 CIR. Its contribution to the model was tested with the data used to examine these discrepancies. Thus, studies with new datasets are needed to investigate further the extent to which this contribution is supported.

## 5. Conclusions

During the first two months of the pandemic in Catalonia, for any given county COVID-19 CIR, homes between 30 and 70 places were more able to avoid COVID-19 infection and preserve lives than larger LTCHs at all levels of county COVID-19 CIR. However, low levels of county COVID-19 CIR proved to protect LTCH residents at LTCHs of up to 150 places. LTCH places located in these counties with low CIR represented no more than 25% of those included in this study, while more than half of the places were in counties with a COVID-19 CIR of 550/100,000 or more. The combination of LTCH size with high community COVID-19 CIRs can be lethal for elderly persons during a public health emergency.

These findings may have broad implications for building new LTCHs and for adaptations to existing ones to provide safer conditions for their residents. The principle of precaution ought to be applied here. The first recommendation of this study is to consider that new LTCHs be designed within the range of 30 to 70 beds and that, where possible, existing ones ought to be divided into self-contained units of 30 to 70 beds. A second recommendation is that more research needs to be done to identify factors associated with failure and relative success. Factors associated with large-sized facilities that led to the high risk of mortality observed in those homes need to be investigated, as do factors associated with the low levels of mortality observed in all LTCHs located in counties with a COVID-19 CIR of less than 250/100,000.

## Figures and Tables

**Table 1:** Distribution of LTCHs by LTCH size, County Population, and County COVID-19 Cumulative Incidence Rate during March–April 2020 in Catalonia (*N* = 965).

	LTCHs		Number of Residents	
	*N*	%	*N*	%
**Total**	965	100%	56,831	100%
**LTCH size**				
<30	298	31%	6544	12%
30–49	201	21%	23,212	41%
50–99	329	34%	7665	13%
100–149	86	9%	10,198	18%
150–199	42	4%	7048	12%
200 and more	9	1%	2164	4%
**County population**				
Barcelona	274	28%	15,477	27%
Between 700,000 and 1,000,000	213	22%	11,644	20%
Between 200,000 and 700,000	181	19%	11,353	20%
Between 100,000 and 200,000	180	19%	11,280	20%
Less than 100,000 inhabitants	117	12%	7077	12%
**County COVID-19 CIR**				
Less than 500/100,000	214	22%	13,640	24%
Between 500 and 750/100,000	154	16%	9386	17%
Between 750 and 900/100,000	263	27%	14,639	26%
Between 900 and 1000/100,000	283	30%	16,005	28%
More than 1000/1,000,000	51	5%	3161	6%

**Table 2:** Distribution of COVID-19 infection and mortality outcomes in LTCHs in Catalonia, March–April 2020, by size, county population, and county COVID-19 CIR and LTCH type.

	COVID-19 Infection in LTCH		Number of LTCH and % with at Least One COVID-19 Death		COVID-19 Deaths (SD)
	*N*	%	*N*	%	Mean (SD)
**Total**	627	65	505	52	7.7 (8.9)
**LTCH size**					
<30	156	52	114	38	3.2 (3.1)
30–49	115	57	87	43	4.1 (3.6)
50–99	236	72	193	59	7.7 (7.0)
100–149	72	84	66	77	11.0 (9.5)
150–199	40	95	38	90	19.3 (13.3)
200 and more	8	89	7	78	30.3 (23.8)
**County population**					
Barcelona	218	80	31	69	8.2 (9.5)
Between 700,000 and 1,000,000	153	72	83	59	7.4 (7.6)
Between 200,000 and 700,000	90	50	77	42	7.5 (9.1)
Between 100,000 and 200,000	118	66	126	46	8.1 (9.9)
Less than 100,000 inhabitants	48	41	188	26	4.9 (6.8)
**County COVID-19 CIR**					
Less than 500/100,000	94	44	56	26	6.6 (8.3)
Between 500 and 750/100,000	83	54	73	46	7.1 (8.4)
Between 750 and 900/100,000	182	69	148	56	7.2 (7.4)
Between 900 and 1000/100,000	225	79	192	68	8.2 (9.5)
More than 1000/100,000	43	84	36	71	9.8 (12.5)
**LTCH Type**					
Generalitat, publicly administered	15	75	14	70	14.4 (10.9)
Generalitat, indirectly administered	33	77	28	65	10.0 (6.6)
Other public institutions, publicly administered	35	58	25	42	8.8 (10.5)
Other public institutions, indirectly administered	15	79	11	58	7.4 (6.4)
For-profit, with rented places	75	76	63	64	9.9 (11.8)
Nonprofit, with rented places	39	81	35	73	11.4 (10.0)
For-profit, completely privately administered	322	61	258	49	6.2 (7.9)
Nonprofit, completely privately administered	93	64	71	49	6.6 (7.6)

## Data Availability

Data are available from the first author upon request.

## Appendix A

**Testing the modification of the association of COVID-19 infection and LTCH size by county COVID-19 CIR and population size.** Using the log-likelihood ratio test (LRT), the interactions of LTCH size with county COVID-19 CIR, population size, and their respective quadratic terms were examined. The hypothesis of no difference between the logistic regression equation without interaction terms and the equation with interaction terms could not be rejected at the 0.05-level (Chi-square = 4.1 with 5 degrees of freedom). Thus, the association between COVID-19 infection and LTCH size did not change with county COVID-19 CIR and county population. Interaction terms were excluded from the model (see Table A1).

**Table A1:** COVID-19 Infection by Size of LTCH: Logistic regression estimates.

	Unadjusted		Adjusted-I		Adjusted-II	
	Coeff.	95% CI	Coeff.	95% CI	Coeff.	95% CI
**Intercept**	−0.324	−0.721–0.181	−2.965	−3.768–2.362	−2.853	−3.581–−2.244
**LTCH size**	0.158	−0.039–0.284	0.237	0.186–0.305	0.241	0.164–0.330
**LTCH size ^2^**	0.002	−0.006–0.018	n.s.	n.s.	n.s.	n.s.
**LTCH type**						
For profit, completely privately administered	--	--	Ref.	Ref.	Ref.	Ref.
Generalitat, publicly administered	--	--	0.156	−0.981–1.702	n.s.	n.s.
Generalitat, indirectly administered	--	--	0.684	−0.146–1.783	n.s.	n.s.
Other public institutions, publicly administered	--	--	0.359	−0.312–1.053	n.s.	n.s.
Other institutions, indirectly administered	--	--	0.424	−0.555–2.615	n.s.	n.s.
For-profit, with rented places	--	--	0.282	−0.293–0.942	n.s.	n.s.
Nonprofit, with rented places	--	--	0.445	−0.317–1.428	n.s.	n.s.
Nonprofit, completely privately administered	--	--	0.202	−0.211–0.626	n.s.	n.s.
**County population**	--	--	0.035	0.011–0.058	0.033	0.010–0.056
**County CIR/100,000**	--	--	0.250	0.173–−0.347	0.241	0.178–0.330
**Goodness of fit**			**Test-1 ^(1)^**		**Test-2 ^(2)^**	
−2 * Log Likelihood	1177.6		1056.7		1061.3	
Degrees of freedom	3		11		4	
Chi square difference of −2 * log likelihoods			120.9 *		4.6	
Degrees of freedom difference			8		7	

^(1)^ Test-1; Testing rejecting the null hypothesis of no difference between the adjusted-I with the unadjusted model; ^(2)^ Test-2: Testing rejecting the null hypothesis of no difference between the adjusted-I with the adjusted_II model; ^2^ Size quadratic; * Significant at the 0.05 level; n.s.—Non significant; CI—confidence interval.

**Testing the modification of the association of absence of COVID-19 death in an LTCH and LTCH size by county COVID-19 CIR and population size**. Interactions of LTCH size with county COVID-19 CIR and population size were tested in the two parts of the hurdle model. In the first part, the logistic regression equation for the absence of death in LTCHs was used to test for the hypothesis of no difference between the regressions without and with interaction terms. Quadratic terms were also considered. This hypothesis could not be rejected at the 0.05-level with an LRT (Chi-square = 7.2 with 5 degrees of freedom). Thus, the association between the absence of COVID-19 death in an LTCH and LTCH size did not change with county COVID-19 CIR and county population.

Interaction terms were excluded from the model (Table A2, Part 1).

**Testing the modification of the association of the number of COVID-19 cases in LTCHs and LTCH size by county COVID-19 CIR and population size**. Interaction terms of LTCH size with county COVID-19 CIR and its quadratic term were significant according to the LTR test (Chi-square = 16.2 with 5 degrees of freedom). However, the 95% confidence intervals of the coefficient for the quadratic term, obtained with a bootstrap procedure with 10,000 replications, included the null hypothesis. The quadratic term was excluded from the hurdle model, part 2. As shown in Table A2, part 2, the LRT test for the interaction of LTCH size with county COVID-19 CIR was significant at 0.05 level. This interaction term was retained in the analysis.

**Table A2:** COVID-19 Mortality in LTCHs with COVID-19 (*N* = 625) by LTCH size: Hurdle Model.

Part 1. Logistic Regression: Absence of COVID-19 Infection by LTCH Size								
	Unadjusted		Adjusted-I		Adjusted-II		Significant Interactions	
	Coeff.	95% CI	Coeff.	95% CI	Coeff.	95% CI	Coeff.	95% CI
**Intercept**	−0.643	−1.402–−0.153	0.897	0.034–1.995	1.097	0.196–1.954	1.097	0.196–2.121
**LTCH size**	−0.138	−0.277–0.141	−0.165	−0.263–−0.097	−0.159	−0.239–−0.107	−0.159	−0.239–−0.096
**LTCH size ^2^**		−0.021–0.008	n.s.	n.s.	n.s.	n.s.	n.s.	n.s.
**LTCH type**								
For profit, completely privately administered	-	-	Ref.	Ref.	Ref.	Ref.	Ref.	Ref.
Generalitat, publicly administered	-	-	−0.727	−12.627–0.799	n.s.	n.s.	n.s.	n.s.
Generalitat, indirectly administered	-	-	0.018	−1.322–0.780	n.s.	n.s.	n.s.	n.s.
Other public institutions, publicly administered	-	-	0.278	−0.656–1.064	n.s.	n.s.	n.s.	n.s.
Other public institutions, indirectly administered	-	-	0.499	−9.201–2.096	n.s.	n.s.	n.s.	n.s.
For profit, with rented places	-	-	0.197	−0.631–0.918	n.s.	n.s.	n.s.	n.s.
Nonprofit, with rented places	-	-	−0.277	−2.044–0.774	n.s.	n.s.	n.s.	n.s.
Nonprofit, completely privately administered	-	-	0.188	−0.465–0.804	n.s.	n.s.	n.s.	n.s.
**County population**	-	-	−0.027	−0.061–0.007	n.s.	n.s.	n.s.	n.s.
**County CIR/100,000**	-	-	−0.144	−0.275–−0.040	−0.2	−0.302–−0.110	−0.200	−0.302–−0.110
**Part 2: Hurdle negative binomial: Number of COVID-19 deaths by LTCH size**								
**Intercept**	0.271	−0.118–0.562	−0.301	−1.003–0.295	−0.369	−1.076–0.102	0.626	−0.475–1.696
**LTCH size**	0.229	0.175–0.313	0.208	0.148–0.296	0.228	0.175–0.299	0.099	−0.038–0.227
**LTCH size ^2^**	−0.004	−0.009–−0.002	−0.003	−0.008–−0.001	−0.004	−0.009–−0.003	−0.004	−0.009–0.001
**LTCH Type**								
For profit, completely privately administered	-	-	Ref.	Ref.	Ref.	Ref.	Ref.	Ref.
Generalitat, publicly administered	-	-	0.422	−0.202–0.877	n.s.	n.s.	n.s.	n.s.
Generalitat, indirectly administered	-	-	0.23	−0.122–0.528	n.s.	n.s.	n.s.	n.s.
Other public institutions, publicly administered	-	-	0.085	−0.358–0.465	n.s.	n.s.	n.s.	n.s.
Other public institutions, indirectly administered	-	-	0.048	−0.664–0.542	n.s.	n.s.	n.s.	n.s.
For profit, with rented places	-	-	−0.236	−0.626–0.086	n.s.	n.s.	n.s.	n.s.
Nonprofit, with rented places	-	-	0.315	−0.059–0.617	n.s.	n.s.	n.s.	n.s.
Nonprofit, completely privately administered	-	-	0.119	−0.259–0.434	n.s.	n.s.	n.s.	n.s.
**County population**	-	-	−0.001	−0.014–0.013	n.s.	n.s.	n.s.	n.s.
**County CIR/100,000**	-	-	0.075	0.005–0.149	0.078	0.019–0.142	−0.035	−0.158–0.078
**Interactions LTCH size**								
**County Population by LTCH size**	-	-	-	-	-	-	-	-
**County CIR by LTCH size**	-	-	-	-	-	-	0.014	0.004–0.030
**Interactions LTCH size ^2^**								
**Population by LTCH size ^2^**	-	-	-	-	-	-	-	-
**CIR by LTCH size ^2^**	-	-	-	-	-	-	-	-
**Dispersion**	1.386	1.030–1.877	1.228	0.884–1.578	1.291	0.960–1.719	1252,000	0.927–1.642
**Goodness of fit**			**Test-1 ^(1)^**		**Test-2 ^(2)^**		**Test-3 ^(3)^**	
−2 * Log Likelihood	3382.1		3338.3		3352.8		3345.2	
Degrees of freedom	7		24		8		9	
Chi squared difference of −2 * log likelihoods			43.8 *		14.5		7.5 *	
Degrees of freedom difference			17		16		1	

^(1)^ Test-1; Testing rejecting the null hypothesis of no difference between the Adjusted-I model with the Unadjusted model; ^(2)^ Test-2: Testing rejecting the null hypothesis of no difference between the Adjusted-I model with the Adjusted_II model; ^(3)^ Test-3: Testing rejecting the null hypothesis of no difference between the interaction model with the Adjusted_II model; ^2^ Size quadratic; * Significant at the 0.05 level; n.s.—Non significant; CI—confidence interval.
